# Effects of Curcumin on the Egg Quality and Hepatic Lipid Metabolism of Laying Hens

**DOI:** 10.3390/ani14010138

**Published:** 2023-12-30

**Authors:** Chenxuan Li, Jiang Gao, Shihui Guo, Bin He, Wenqiang Ma

**Affiliations:** 1 Key Laboratory of Animal Physiology and Biochemistry, Ministry of Agriculture and Rural Affairs, College of Veterinary Medicine, Nanjing Agricultural University, Nanjing 210095, China; 2022207005@stu.njau.edu.cn (C.L.); 2022007052@stu.njau.edu.cn (J.G.); 2017107012@njau.edu.cn (S.G.); heb@njau.edu.cn (B.H.); 2 MOE Joint International Research Laboratory of Animal Health & Food Safety, Nanjing Agricultural University, Nanjing 210095, China

**Keywords:** curcumin, egg quality, hepatic lipid metabolism, hens

## Abstract

**Simple Summary:**

The poultry industry is increasingly seeking natural, safe, and cost-effective feed additives, due to the ban on antibiotics and rising production demands. Curcumin, a natural plant extract, has demonstrated antioxidant and anti-inflammatory properties in feed additive studies. However, the mechanism by which curcumin supplementation improves the performance and health status of laying hens needs to be more thoroughly investigated. In this study, our results showed that a 200 mg/kg curcumin supplementation significantly enhanced egg quality by targeting and regulating oviduct expansion glycoprotein gene expression. Additionally, curcumin improved hepatic lipid metabolism in laying hens by regulating hepatic fatty acid synthesis genes. These findings provide a basis for considering curcumin as a valuable feed additive in the laying hen industry.

**Abstract:**

Curcumin, the major active compound of turmeric, has shown potential benefits for poultry health and production in various studies. However, its specific role in enhancing the egg quality and liver health of laying hens, as well as its underlying mechanisms, have yet to be determined. Here, a total of 600 Su Qin No.1 Laying hens, aged 55 weeks and with similar laying rates, were randomly placed into five groups, with 10 replicates of 12 hens each. Curcumin doses of 0, 100, 200, 400, and 800 mg/kg were added to the basal diet to form the experimental groups. After an 8-week feeding period, no significant changes were observed in the production performance of laying hens due to curcumin supplementation. However, additional tests revealed that a 200 mg/kg curcumin supplementation improved albumen height, yolk color, Haugh unit, and eggshell thickness, while reducing the thin albumen’s weight and proportion. This was accompanied by a significant down-regulation of the mRNA expression level of the Prolactin Receptor (*Prlr*) in the oviduct magnum. Furthermore, the number of hepatic lipid droplets and the hepatic triglyceride (TG) content, as well as malondialdehyde (MDA) levels were significantly reduced, indicating improved hepatic lipid metabolism and oxidative status. This was accompanied by a significant reduction in the expressions of sterol regulatory element binding protein-1 gene (*Srebp-1*), fatty acid synthase gene (*Fasn*), as well as fatty acid synthase (FASN), which are closely related to fatty acid synthesis in the liver. Overall, these findings suggest that curcumin supplementation at a dosage of 200 mg/kg could lead to significant improvements in egg quality and hepatic lipid metabolism.

## 1. Introduction

High egg production demands considerable energy from laying hens during egg-laying periods. The hefty energy consumption can overburden the liver, leading to disorders of fat metabolism. Caged hens with limited movement are prone to excess energy and lipid peroxidation that can damage the liver through diseases such as fatty liver hemorrhage syndrome (FLHS) [1,2]. FLHS is characterized by elevated hepatic triglyceride levels accompanied by hepatic hemorrhaging and substantial intra-abdominal lipid build-up. This condition can result in mass mortality during peak egg production due to internal bleeding from liver rupture [3], causing significant economic losses for the laying hen industry. It provides both safety and efficiency benefits to supplement feed with plant extracts, which can reduce the hepatic fat accumulation in laying hens.

Egg quality depends on factors like the health of the laying hens and feed additives [4,5,6,7]. It encompasses eggshell quality, egg white quality, and egg yolk quality. The oviduct plays a key role in egg formation and development, including the eggshell, albumen, and yolk. The shell gland or uterus forms the eggshell [8], while the magnum section of the oviduct produces albumen, which is evaluated based on its thickness, firmness, and clarity [9]. The yolk develops in the ovary and moves through the infundibulum into the oviduct, and its quality relies on its size, color, and nutritional content [10]. Numerous plant extracts have been found to improve egg quality by enhancing the oviduct health of laying hens [11,12,13,14]. Research on thyme [15] and rosemary [16] extracts have demonstrated their antimicrobial and antioxidant properties, indicating a positive impact on egg quality.

Curcumin, extracted from turmeric, exhibits antioxidant, anti-inflammatory, antibacterial, and immunomodulatory properties [17,18,19]. It is documented that curcumin can improve intestinal health [20,21], oviduct health [22], and fat deposition [23,24] in poultry. In laying hens, supplementing 30 or 50 mg/kg of curcumin enhanced egg quality and anticoccidiosis effects [25]. A study by Liu et al. [22] found that 150 mg/kg of curcumin improved eggshell thickness, strength, and albumen height in heat-stressed hens. Khan et al. [26] noted that 0.5% turmeric powder, containing primarily curcumin, increased egg production and body weight, while 1.0% turmeric significantly increased yolk weight and index. Curcumin has been shown to reduce abdominal and liver fat in broilers [23] and laying hens [24] by affecting the expression levels of the genes involved in lipogenesis and lipolysis. However, studies on the mechanisms of curcumin’s effects on laying hens are limited, and further investigation is required to clarify these behaviors. Determining how curcumin modulates egg quality and lipid metabolism could optimize its role in poultry production and health.

The objective of this study is to elucidate the impacts of a suitable supplementary dose of curcumin on the productivity, quality of eggs, and lipid metabolism of laying hens, as well as its governing mechanism. This will establish a theoretical foundation for the utilization and implementation of curcumin in the egg production of laying hens.

## 2. Materials and Methods

### 2.1. Animals, Experimental Design, and Sampling

A total of 600 55-week-old Su Qin No.1 laying hens were randomly divided into 5 groups, each having 10 replicates of 12 hens. Battery cages (60 × 45 × 43 cm; length × width × height), with six hens per cage, were used in the experiment. Different doses of curcumin (Xinlu Biotechnology Co., Xian, China) were added to the basal diet: 0, 100, 200, 400, and 800 mg/kg, to form distinct experimental groups. Following a 14-day period of adjusting to the diet, the official experimental period of 8 weeks commenced. The composition was provided and the nutritional levels of the basal diet for Su Qin No.1 have been determined through analysis (see Table 1). To provide a high-quality environment for the laying hens, the indoor temperature (25–30 °C) and humidity (50–70%) were kept constant during feeding. The daily light duration was automatically controlled to 16 h (05:00–21:00). During the feeding period, the hens were fed regularly and quantitatively every day, i.e., 100 g feed/hen was given at the beginning of the daily light phase (05:00) and water was freely available. The remaining feed was weighed and recorded every Sunday before the start of the light period to calculate the feed intake.

At the end of the 8-week experiment, based on a statistical analysis of the production performance and egg quality of the hens in each group during the previous period, we decided to sample the control and 200 mg/kg curcumin-supplemented groups of laying hens for further testing. A total of 15 hens from each group were selected at random, weighed, and sacrificed after anesthesia. The liver and magnum oviduct were extracted, rinsed with saline solution, and dried by blotting with filter paper. The molecular samples were placed in 2 mL EP tubes, immersed in liquid nitrogen, and transferred to a −80 °C refrigerator. The tissue samples were placed in EP tubes filled with polyformaldehyde and stored in cold storage at 4 °C.

### 2.2. Production Performance

Throughout the experiment, a daily recording of egg production in terms of quantity and weight was conducted for each replicate, whereas feed consumption was recorded on a weekly basis. The calculation of feed conversion involved determining the ratio between the average amount of feed consumed and the average weight of eggs produced.

### 2.3. Detection of Egg Quality

Egg quality was evaluated every two weeks by selecting 30 eggs from each group. Eggshell strength was evaluated using an eggshell strength tester (WW-2A, Nanjing Soil Instrument Factory Co., Ltd., Nanjing, China). To determine the thickness of the eggshell (excluding the membrane), a spiral micrometer was employed to measure the size of the big end, small end, and middle section. The average of these three measurements was considered the shell thickness. A multifunctional egg quality analyzer (EMT-5200, Robotmation Co., Ltd., Tokyo, Japan) was utilized to measure various indicators of egg quality, such as egg weight, egg white height, Haugh units, and yolk color. After the tests, eggs were weighed for their shell, egg albumen, and yolk separately. The eggshell index, albumen index, and yolk index were calculated by determining the ratios of the weight of the eggshell, albumen, and yolk to the weight of the egg.

### 2.4. Detection of Thick and Thin Albumen

On the same basis as sampling, 30 eggs were randomly selected from the control group and the experimental group with a 200 mg/kg curcumin supplementation. The eggs were opened, and the contents, excluding the shell, were weighed. The egg contents were strained using an egg filter to remove the yolk. The remaining albumen was passed through a 40-mesh test sieve and left for 2 min. The thin albumen, which filtered through the test sieve, was weighed, while the thick albumen left on the test sieve was also weighed. The ratios of the weight of the thick albumen or thin albumen to the weight of the contents were calculated to obtain the thick albumen index and thin albumen index, respectively.

### 2.5. Determination of Triglyceride Content and Antioxidant Index in the Liver

Triglyceride levels were measured using the Triglyceride Assay Kit (A110-1-1, Nanjing Jiancheng Bioengineering Institute, Nanjing, China) following the manufacturer’s instructions. Liver tissues were accurately weighed and homogenized in ice water bath conditions with saline at a weight–volume ratio of 1 g: 9 mL. The supernatant was collected after centrifugation at 2500 rpm for 10 min for measurement. Liver antioxidant indexes were measured using commercial kits from Nanjing Jiancheng Bioengineering Institute and according to their instructions: the total superoxide dismutase (SOD) test kit (A001-1), malondialdehyde (MDA) test kit (A003-1), catalase assay (CAT) test kit (A007-2), and glutathione peroxidase (GSH-Px) test kit (A005-1).

### 2.6. Microscopic Examination of Liver Sections

Liver samples fixed in a 10% formaldehyde solution were dehydrated and embedded in paraffin. HE staining was used to observe the cellular structure of liver tissue. The sections were deparaffinized with xylene, dehydrated with alcohol, and then stained with hematoxylin and eosin. Intrahepatic adipose tissue was observed using oil red staining. The sections were then rinsed with distilled water and immersed in hematoxylin for light staining for 1–2 min. After rinsing with distilled water and 70% ethanol for 5–10 s, the sections were immersed in Sudan red III staining solution and placed in a thermostat for 15–30 min. Finally, the sections were sealed with glycerol gelatin and stored at −20 °C.

### 2.7. Expression Analysis of Genes

Total RNAs were extracted from the liver and oviduct magnum using TRIzol reagent (1559602, Invitrogen, Carlsbad, CA, USA) according to the manufacturer’s instructions. The RNA’s purity and concentration were assessed using a spectrophotometer (Nanodrop ND-1000, Thermo Scientific, Waltham, MA, USA). A total of 1 μg of RNA was converted into complementary DNA using the reverse transcription kit (R132-01, Vazyme Biotech Co., Ltd., Nanjing, China). The cDNA samples were identified using the SYBR Green PCR Master Mix Kit (Roche, Basel, Switzerland). An ABI 7500 Real-time Detection System (Applied Biosystems, Carlsbad, CA, USA) was utilized to perform RT-qPCR. The mRNA levels were computed and standardized using the 2^−ΔΔCT^ technique relative to β-actin or 18S. Singke Biotechnology Co., Ltd. (located in Beijing, China) synthesized the primers, and the primer sequences for RT-qPCR can be found in Table S1.

### 2.8. Expression Analysis of Proteins

Protein was extracted from frozen liver samples weighing 50 mg, and the BCA Protein Assay kit (NO.23227, Thermos Scientific, Waltham, MA, USA) was used to measure protein concentrations following the instructions provided by the manufacturer. In total, 50 μg of protein was loaded per lane for electrophoresis on a 10% SDS-PAGE gel. After transferring the protein onto a nitrocellulose filter membrane, it was blocked in a 4% milk solution and incubated with primary antibodies (FASN, AB43451, 1:1000, AbSci, MD, USA; SREBP1, 14088-1-AP, 1:1000, ThermoFisher, Waltham, MA, USA) and secondary antibodies (111-095-003, Jackson ImmunoResearch, West Grove, PA, USA). For loading control, β-Tubulin (BS1699, Bioworld, Bloomington, IN, USA) was employed.

### 2.9. Statistical Analysis

All data are expressed as means ± SEM. Data were analyzed using a One-Way ANOVA and *t*-test. Orthogonal polynomial contrasts were used to test the linear or quadratic nature of the response to incremental concentrations of curcumin. Significant differences were determined using IBM SPSS Statistics 20(IBMCorp., Armonk, NY, USA), and a significance level of *p* < 0.05 was applied.

## 3. Results

### 3.1. Production Performance

As shown in Table 2, curcumin supplementation did not significantly affect the production performance of laying hens. The laying rate, average feed consumption, average egg weight, and feed-to-egg ratio in laying hens remained unchanged over the 8-week period when curcumin was supplemented at doses ranging from 100 to 800 mg/kg (*p >* 0.05).

### 3.2. Egg Quality

Although the addition of 100 mg/kg of curcumin did not show significant effects, higher doses of curcumin achieved the expected results (Table 3). With a 200 mg/kg curcumin supplementation, the albumen height and eggshell thickness increased linearly (*p* < 0.05 or *p* < 0.01), accompanied by a significant linear and quadratic increase in the Haugh unit (*p* < 0.05). The addition of curcumin at both 400 mg/kg and 800 mg/kg resulted in a significant linear improvement in the color of the egg yolk (*p* < 0.05). Additionally, the 800 mg/kg curcumin supplementation also significantly linearly improved eggshell thickness (*p* < 0.01).

### 3.3. Albumen Quality and Magnum’s Glycoprotein Expression

**Figure 1:**
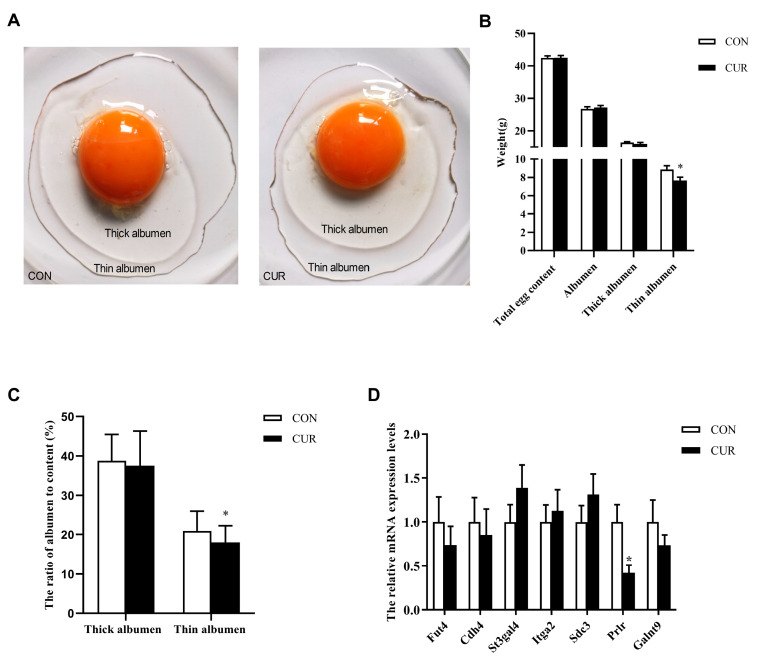
Effects of curcumin on albumen quality and glycoprotein expression in the magnum of laying hens. (**A**) Thick and thin egg albumen between two groups. (**B**) Weight of egg contents, total albumen, thick albumen, and thin albumen across two groups. (**C**) The ratios of thick and thin albumen to egg content between two groups. (**D**) Expression levels of genes related to glycoprotein metabolism in the oviduct magnum of laying hens across two groups. CON = control diet group; CUR = control diet supplemented with 200 mg/kg curcumin. 18s RNA was used as a reference. Data are presented as mean ± SEM; n = 8 (**A**–**C**), or 7 (**D**). * indicated significantly different expression as *p* < 0.05.

In Figure 1A–C, the addition of curcumin at the dose of 200 mg/kg did not result in notable alterations in the weight of the total contents, albumen, or thick albumen. Nevertheless, the weight of the thin albumen and the ratio of its content was significantly reduced (*p* < 0.05). The addition of curcumin led to a notable decrease in the expression of *Prlr* (*p* < 0.05), a gene associated with glycoprotein metabolism in the oviduct magnum, as indicated by the RT-PCR findings (Figure 1D).

### 3.4. Liver Lipid Metabolism and Antioxidant Index

**Figure 2:**
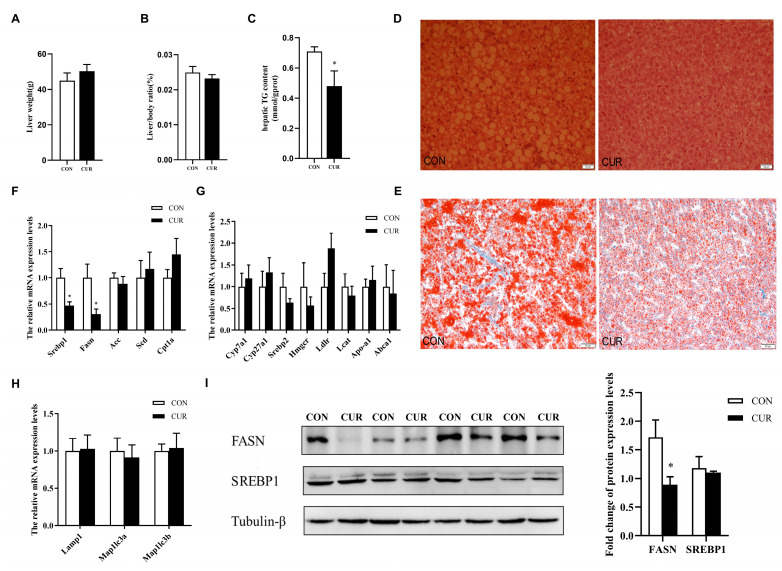
Effect of curcumin on liver lipid metabolism in laying hens. (**A**) The liver weight in laying hens. (**B**) The liver-to-body ratio. (**C**) Hepatic TG content. (**D**) Liver sections stained with HE (40×) in laying hens. (**E**) Liver sections stained with oil red (10×) in laying hens. (**F**) Hepatic expression levels of fatty acid synthesis and β-oxidation-related genes in laying hens. (**G**) Hepatic expression levels of cholesterol transport, synthesis, and degradation-related genes in laying hens. (**H**) Hepatic expression levels of lipid autophagy-related genes in laying hens. (**I**) Hepatic expression levels of fatty acid synthesis-related proteins (FASN and SERBP1) in laying hens. β-actin was used as a reference in (**F**–**H**), and β-tubulin was used as a reference in (**I**). CON = control diet group; CUR = control diet supplemented with 200 mg/kg of curcumin. Data are presented as mean ± SEM, n = 14 (**A**–**C**), 5 (**D**,**E**), or 6 (**F**–**I**). * indicated significantly different expression as *p* < 0.05.

No differences were observed between the two groups in terms of liver weight and the ratio of the liver to the body, as shown in Figure 2A,B. However, curcumin supplementation led to a significant reduction in liver triglyceride (TG) levels (*p* < 0.05). Fewer lipid droplets were observed in the liver tissue of laying hens in the curcumin-supplemented group than in the control group, as depicted in Figure 2D,E. Curcumin also significantly decreased the liver expression of genes associated with fatty acid synthesis, such as sterol regulatory element binding protein-1 (*Srebp1*) and fatty acid synthase (*Fasn*) in the liver of laying hens (Figure 2F, *p* < 0.05). The expression of genes related to cholesterol transport, synthesis, degradation (Figure 2G), and lipid autophagy (Figure 2H) in the liver of laying hens did not show any notable change. Additional findings revealed that the hepatic FASN, a protein responsible for synthesizing fatty acids, was substantially suppressed in the laying hens that were administered curcumin (*p* < 0.05). On the other hand, there were no notable differences in the expression level of SERBP1 (*p* > 0.05). In addition, curcumin supplementation had no significant effect on liver SOD, CAT, and GSH-Px levels, but did significantly reduce hepatic MDA levels (see Table S2).

## 4. Discussion

This study investigated the additional effects of curcumin on the egg quality and liver lipid metabolism of laying hens. The findings revealed that the inclusion of 200 mg/kg of curcumin in their diet enhanced eggshell thickness and albumen height, and reduced thin albumen content and hepatic triglyceride accumulation in laying hens. These results suggest that curcumin supplementation can improve the egg quality and liver health of laying hens.

Studies have revealed that curcumin has the ability to enhance the feed intake, weight gain, and feed conversion rate in broiler chickens [27,28], as well as enhance production performance in laying hens [22,29,30]. Turmeric powder, which contains the active ingredient curcumin, has been reported to significantly improve and maintain higher egg production in old laying hens when added at levels of 0.1%, 0.25%, 0.5%, 1%, 2%, or 4% [29,30]. Additionally, Liu et al. [22] found that the inclusion of 100 mg/kg or 150 mg/kg of curcumin in the diet greatly increased egg production in heat-stressed laying hens. In our study, supplementation with 100–800 mg/kg of curcumin did not significantly influence the feed intake, laying rate, or egg weight of laying hens. This is consistent with previous research showing that a curcumin addition did not result in changes in feed consumption, egg production, or egg weight [25,31]. Furthermore, the supplementation of 30 and 50 mg/kg of curcumin had no influence on the production performance of laying hens [25]. da Rosa et al. [31] also observed that the addition of 200 mg/kg of curcumin had no influence on egg production in laying hens. One factor that negatively affects egg production is oxidative stress, which can be induced by heat exposure [32] or aging in laying hens [33,34]. Since curcumin acts as an antioxidant, it plays an essential role in suppressing oxidative stress and improving the laying rate in aged hens and heat-stressed hens.

The quality of eggs can be evaluated using key indicators such as egg yolk color, eggshell quality, and albumen quality. Previous studies have found that adding curcumin significantly improves yolk color, which is an important factor consumers consider when judging egg quality [25,26,29]. Our results are in agreement with these findings, as curcumin at 400 mg/kg and 800 mg/kg noticeably boosted color levels. In addition, the 200 mg/kg and 800 mg/kg curcumin additions also amplified shell thickness, which aligns with earlier research findings [22,35]. Scientists propose that curcumin’s antioxidant properties improve the uterine microenvironment, where most calcium deposition occurs, optimizing the quality and thickness of the shell. The Haugh unit characterizes freshness and albumen quality, with higher values corresponding to taller albumen and fresher eggs [36,37,38]. In the present study, adding 200 mg/kg of curcumin increased the Haugh unit and albumen height. Albumen is mainly produced in the oviduct magnum and its main component is glycoprotein. Albumen can be divided into thick and thin types depending on ovomucin levels. This study found that 200 mg/kg of curcumin did not significantly change the total or thick albumen amounts, but it did reduce the thin albumen’s weight and proportion. A study of the RNA-Seq revealed that the low expression of magnum prolactin receptor (*Prlr*) was associated with a high percentage of thick albumen in eggs [39]. In laying hens, *Prlr* mRNA is widely present in the reproductive and intestinal organs, as well as in the oviduct. Prolactin decreases and boosts the luteinizing hormone, shortening laying intervals to enhance the laying performance [40]. In the current study, further analysis showed that curcumin down-regulated oviduct *Prlr* gene expression. Therefore, the *Prlr* gene exhibited a decrease in expression in the hens fed curcumin, indicating that a reduction in *Prlr* gene activity in the oviduct could potentially hinder the accumulation of thin protein.

Laying hens require a high calorie intake due to the traits of egg production, but their limited activity leads to a daily surplus. The liver is responsible for lipid metabolism in hens, and excess peroxides can induce lipid peroxidation, protein oxidation, and enzyme inhibition, overwhelming cellular homeostasis and ultimately leading to severe fatty liver syndrome [2,41]. Studies have shown that curcumin can decrease hepatic lipid accumulation and enhance the antioxidant capacity of broiler chickens [23] and laying hens [24]. In a study on broilers, curcumin was found to decrease TG concentrations and modulate lipid metabolism, reducing accumulation while maintaining hepatocyte morphology [23]. Additionally, supplementing laying hens with 150 mg/kg of curcumin resulted in a decrease in hepatic TG content, an improvement in antioxidant enzyme activities, and a reduction in MDA levels [24]. This suggests that curcumin functions as an antioxidant to alleviate stress and enhance the liver health of laying hens.

Additionally, curcumin lessens hepatic fat deposits by regulating the gene expression involved in lipogenesis and lipolysis, such as acetyl-CoA carboxylase (*Acc*), fatty acid synthase (*Fsan*), sterol regulatory element binding transcription factor 1c (*Srebp-1c*), ATP citrate lyase (*Acly*), peroxisome proliferator-activated receptor α (*Ppar α*) and carnitine palmitoyltransferase 1 (*Cpt-1*) [23,24]. In laying hens, FASN plays a crucial role in the metabolism of fatty acids and the synthesis of lipids [42], with palmitic acid being its primary product. It is documented that adding 200 mg/kg of curcumin greatly decreases FASN enzyme activity to lower lipid deposits in broilers [43]. Our results also proved that curcumin decreased fatty acid synthesis protein FASN expression in the liver of laying hens. Additionally, the liver plays a crucial role in synthesizing yolk precursors during egg production, producing significant amounts of apolipoproteins and vitellogenin (VTG), the primary components of egg yolk protein. These substances transport and deposit additional nutrients into the ovary, thereby regulating rapid follicle development [44]. As the antioxidant level in the liver of laying hens decreases with age, this can result in a reduction in the production of yolk precursors [45]. Thus, liver health is closely linked to egg quality, and particularly yolk quality. The findings are consistent with the results of this study, suggesting that curcumin supplementation can improve the liver health and egg quality of laying hens.

In summary, the addition of curcumin improves egg quality and reduces hepatic lipid deposition in laying hens. Curcumin supplementation enhances the color of egg yolks, increases albumen height, and decreases the amount of thin albumen by lowering the expression of the glycoprotein metabolism-associated gene *Prlr* in the oviduct magnum. Moreover, the decline in liver lipid accumulation in laying hens is attributed to the curcumin-induced reduction of FASN expression, a protein involved in fatty acid synthesis. The hens’ improved liver health status may further contribute to enhancing egg quality.

## 5. Conclusions

In our research, we discovered that curcumin effectively targets and regulates the expression of oviduct magnum glycoprotein genes in laying hens, leading to improved egg quality. Furthermore, curcumin supplementation optimizes the status of hepatic lipid metabolism by regulating hepatic fatty acid synthesis genes. Given the urgent need for cost-effective, safe, and natural feed additives for the poultry industry, our study provides a solid theoretical foundation for the application of curcumin in the layer industry.

## Figures and Tables

**Table 1:** Composition and nutrient level of basal diet for laying hens.

Items	Content (%)
Ingredients	
Corn	65.00
Soybean meal	22.00
Limestone	8.00
Premix ^1^	5.00
Total	100.00
Nutrient levels	
Metabolizable energy (Mcal/kg)	2.62
Crude protein	14.80
Lysine	0.75
Methionine	0.32
Methionine + cysteine	0.56
Threonine	0.57
Isoleucine	0.60
Calcium	3.87
Total phosphorus	0.50
Available phosphorus	0.26

^1^ 5% laying hen premix is 5% per kilogram of diet. Formula: vitamin A, 10,800 IU; vitamin D3, 2700 IU; vitamin E, 27 mg; vitamin K, 0.84 mg; vitamin B1, 0.72 mg; vitamin B2, 5.4 mg; vitamin B3, 9 mg; vitamin B5, 36 mg; vitamin B6, 2.7 mg; vitamin B11, 0.24 mg; vitamin B12, 0.009 mg; Biotin, 0.09 mg; Cu, 8 mg; Fe, 100 mg; Mn, 100 mg; Zn, 100 mg; Se, 0.3 mg.

**Table 2:** Effects of curcumin supplementation on production performance of laying hens.

Items	Curcumin Supplementation (mg/kg)					SEM ^1^		*p*-Value	
	0	100	200	400	800		Treatment	Linear	Quadratic
1–4 weeks									
Laying rate (%)	60.33	58.22	60.07	59.30	56.83	0.03	0.704	0.345	0.657
Average feed intake (g)	97.33	95.17	96.29	93.99	94.44	2.17	0.541	0.164	0.758
Average egg weight (g)	49.87	49.83	50.02	49.51	49.58	0.32	0.485	0.220	0.586
Feed/egg ratio (g/g)	1.95	1.91	1.93	1.90	1.91	0.04	0.727	0.275	0.631
5–8 weeks									
Laying rate (%)	59.20	58.95	60.47	60.71	56.66	0.03	0.595	0.593	0.227
Average feed intake (g)	97.9	96.14	97.18	96.25	95.83	1.48	0.590	0.218	0.828
Average egg weight (g)	49.06	48.77	48.87	48.99	48.73	0.29	0.770	0.499	0.919
Feed/egg ratio (g/g)	2.00	1.97	1.99	1.96	1.97	0.03	0.729	0.300	0.869
1–8 weeks									
Laying rate (%)	59.76	58.58	60.25	60.01	56.74	0.03	0.661	0.440	0.390
Average feed intake (g)	97.64	95.65	96.73	95.11	95.13	1.77	0.549	0.172	0.780
Average egg weight (g)	49.54	49.40	49.53	49.27	49.23	0.27	0.682	0.224	0.809
Feed/egg ratio (g/g)	1.97	1.94	1.95	1.93	1.93	0.03	0.713	0.274	0.707

*p* > 0.05. ^1^ SEM, standard error of means.

**Table 3:** Effects of curcumin supplementation on egg quality of laying hens.

Items	Curcumin Supplementation (mg/kg)					SEM ^1^	*p*-Value		
	0	100	200	400	800		Treatment	Linear	Quadratic
Shell weight (g)	6.90	6.71	6.64	6.58	6.68	0.24	0.743	0.292	0.370
Shell index (%)	13.87	13.52	13.20	13.06	13.46	0.00	0.372	0.181	0.140
Yolk weight (g)	16.20	16.48	16.53	16.60	16.25	0.45	0.872	0.827	0.299
Yolk index (%)	32.48	33.19	32.85	32.88	32.71	0.01	0.842	0.910	0.399
Albumen weight (g)	26.80	26.51	27.23	27.27	26.73	0.74	0.811	0.7040	0.546
Albumen index (%)	53.65	53.29	53.96	54.06	53.83	0.01	0.858	0.500	0.879
Albumen height (mm)	5.00 ^b^	5.66 ^ab^	6.04 ^a^	5.12 ^b^	5.73 ^ab^	0.39	0.044	0.033	0.175
Yolk color score	12.20 ^b^	12.47 ^b^	12.61 ^b^	12.73 ^a^	13.18 ^a^	0.29	0.015	0.001	0.677
Haugh unit	72.80 ^b^	76.64 ^ab^	80.61 ^a^	72.34 ^b^	74.29 ^b^	3.03	0.046	0.022	0.048
Shell breaking strength (kg/cm^2^)	3.39	3.22	3.23	3.29	3.17	0.23	0.912	0.479	0.830
Shell thickness (mm)	0.31 ^B^	0.30 ^B^	0.33 ^A^	0.31 ^B^	0.33 ^A^	0.01	0.000	0.000	0.563

Different lowercase letters in the shoulder label indicate a significant difference (*p* < 0.05), and different uppercase letters indicate a very significant difference (*p* < 0.01). ^1^ SEM, standard error of means.

## Data Availability

All relevant data are within the manuscript.

## Supplementary Materials

The following supporting information can be downloaded at https://www.mdpi.com/article/10.3390/ani14010138/s1, Table S1: primer sequence of the target genes. Table S2: effects of curcumin on liver antioxidant indexes.
