# Supplementary material for: Effects of Curcumin on the Egg Quality and Hepatic Lipid Metabolism of Laying Hens
Source: Animals (Basel). 2023 Dec 30;14(1):138. doi: 10.3390/ani14010138 (PMC10778355; doi:10.3390/ani14010138)
Supplement: Supplementary file 1 [file animals-14-00138-s001.zip › animals-2756290-supplementary.pdf]

Table S1. Primer sequence of the target genes

Target gene	Forward sequence (5′-3′)	Reverse sequence (5′-3′)
18s RNA	GAAACGGCTACCACATCCA	CACCAGACTTGCCCTCCA
Prlr	ATCCAACCATTCTCTTCTCCT	CACTCTGTCCAACATTCCTG
Fut4	CGCCGACTCCTTCATCCA	CAGTAATGCTCATCCCAGAAAGAC
Cdh4	CTGCTCCACCACCACTCT	TGCTGCTCGCTTACCTTCTA
St3gal4	TGAAGCAGCCAAGGAAGGT	AGGTCGCAGAAGTGGAGAG
Itga2	ATTGGTGTTGTGATTGGCAGTAT	CAGTCTTTGTCTTTCGTGAGTTCT
Sdc3	CATCAGCAACAGGACACAGT	GGAGCAGGAGCCAAGAAG
Galnt9	TTACACAGAACGGTCCAATCATC	CTCAGCCTCAGCAGTCAATG
β-actin	TGCGTGACATCAAGGAGAAG	TGCCAGGGTACATTGTGGTA
Acc	GCCTCCGAGAACCCAA	CCAGCAGTCTGAGCCACTA
Cpt1a	GGCATTGACCGCCATCTGT	GAAACACCGTAACCATCATCAGC
Fasn	CGTCATCACCGTCTATC	GTAGGCTCCTCCCATC
Lamp1	ACACTTCTTTCTTCCCC	CGCTACCATCACTTTTC
Map1lc3a	TGTAGGGCGGTCAATC	ACGGGAACATAGCAAATA
Map1lc3b	CCGCACCTTCGAGCAA	GGAGATGGGCGTGGAGA
Scd	CTATGCGGGGCTACTT	GGCTGGCTGGAATGAA
Srebp1	CTACCGCTCATCCATCAACG	CTGCTTCAGCTTCTGGTTGC
Srebp2	CCCAGAACAGCAAGCAAGG	GCGAGGACAGGAAAGAGAGTG
Hmgcr	TTGGATAGAGGGAAGAGGGAAG	CCATAGCAGAACCCACCAGA
Cyp7a1	CATTCTGTTGCCAGGTGATGTT	GCTCTCTCTGTTTCCCGCTTT
Cyp27a1	AGGACTTTCGTCTGGCTCT	CTCCGCATCGGGTATTT
Abca1	TCCTCTGGCTTAGACTTGA	CTCGTAGTTGTATTCGGTAA
Apo-a1	GTGACCCTCGCTGTGCTCTT	CACTCAGCGTGTCCAGGTTGT
Lcat	CTGGTGAACAACGGCTACG	GTGCCCAATGAGGAAGACA
Ldlr	CCACCATTTGGCAGAGGAA	ACCGCAGTCAGACCAGAAGAG

Table S2. Effect of curcumin on liver antioxidant indexes

**Item**	**Curcumin supplementation (mg/kg)**		***p*-value**
	**0**	**200**	
SOD (U/mgprot)	6.89±0.24	7.99±0.58	0.09
CAT (U/mgprot)	63.32±7.29	66.72±5.95	0.72
MDA (nmol/mgprot)	17.04±4.13[a]	6.77±1.40[b]	0.03
GSH-Px (U/mgprot)	73.27±3.71	79.88±10.12	0.54

Data are presented as Mean ± SEM, Different lowercase letters in the shoulder label indicate a significant difference ($p < 0.05$).
